# Tracing strategic divergence: archetypal and counterfactual analysis of StarCraft II gameplay trajectories

**DOI:** 10.3389/frai.2025.1724493

**Published:** 2026-01-06

**Authors:** Jie Zhang, Weilong Yang

**Affiliations:** Academy of Military Science, Beijing, China

**Keywords:** analytical framework, archetypal path analysis, counter factual alignment, dimensionless deviation metric, process-level interpretability, StarCraft II

## Abstract

**Introduction:**

To address the challenges of data heterogeneity, strategic diversity, and process opacity in interpreting multi-agent decision-making within complex competitive environments, we have developed TRACE, an end-to-end analytical framework for StarCraft II gameplay.

**Methods:**

This framework standardizes raw replay data into aligned state trajectories, extracts “typical strategic progressions” using a Conditional Recurrent Variational Autoencoder (C-RVAE), and quantifies the deviation of individual games from these archetypes via counterfactual alignment. Its core innovation is the introduction of a dimensionless deviation metric, |Δ|, which achieves process-level interpretability. This metric reveals “which elements are important” by ranking time-averaged feature contributions across aggregated categories (Economy, Military, Technology) and shows “when deviations occur” through temporal heatmaps, forging a verifiable evidence chain..

**Results:**

Quantitative evaluation on professional tournament datasets demonstrates the framework’s robustness, revealing that strategic deviations often crystallize in the early game (averaging 8.4% of match duration) and are frequently driven by critical technology timing gaps. The counterfactual generation module effectively restores strategic alignment, achieving an average similarity improvement of over 90% by correcting identified divergences. Furthermore, expert human evaluation confirms the practical utility of the system, awarding high scores for Factual Fidelity (4.6/5.0) and Causal Coherence (4.3/5.0) to the automatically generated narratives.

**Discussion:**

By providing openaccess code and reproducible datasets, TRACE lowers the barrier to large-scale replay analysis, offering an operational quantitative basis for macro-strategy understanding, coaching reviews, and AI model evaluation.

## 1 Introduction

Complex multi-agent environments have emerged as critical testbeds for advancing artificial intelligence research, offering unique challenges in decision-making, strategic planning, and competitive dynamics. Among these environments, real-time strategy (RTS) games, particularly StarCraft II, have established themselves as canonical benchmarks due to their vast state-action spaces, imperfect information constraints, real-time decision requirements, and the necessity for both micro-level tactical control and macro-level strategic planning (Vinyals et al., 2019). The game’s complexity, which surpasses traditional board games by several orders of magnitude, provides an ideal platform for studying emergent behaviors, strategic diversity, and the intricate interplay between short-term tactics and long-term strategy (Ontañón et al., 2013).

The development of superhuman AI agents has marked significant milestones in this domain. DeepMind’s AlphaStar achieved Grandmaster level performance, ranking above 99.8% of active players on Battle.net across all three races, demonstrating the potential of deep reinforcement learning combined with self-play and imitation learning (Vinyals et al., 2019). Similarly, systems like SCC have shown that efficient architectures can achieve competitive performance with significantly reduced computational resources (Wang et al., 2020). However, despite these remarkable achievements in performance metrics, a fundamental challenge persists: the “black-box” nature of these sophisticated neural network architectures obscures the underlying strategic principles and decision-making processes that drive their success (Barredo Arrieta et al., 2020). This opacity creates a critical gap between achieving superhuman performance and generating interpretable insights that could benefit player training, strategic understanding, and the broader development of explainable AI systems.

To address these multifaceted challenges, this paper introduces TRACE (Trajectory Analysis and Counterfactual Explanation), a comprehensive analytical framework designed to bridge the gap between raw gameplay data and interpretable strategic insights. Our framework advances the state-of-the-art in several key dimensions. First, it establishes a unified data processing pipeline that transforms heterogeneous replay data into standardized, temporally aligned trajectory representations, enabling systematic cross-game and cross-tournament comparisons. Second, it leverages archetypal path analysis to automatically discover and characterize typical strategic progressions without requiring predefined strategy labels or extensive domain knowledge. Third, and most innovatively, it introduces a counterfactual alignment paradigm that quantifies strategic deviation through a novel dimensionless metric |*Δ*|, providing process-level interpretability by revealing both which game elements (economy, army composition, technology choices) are most critical and when significant deviations occur during gameplay.

The introduction of the deviation metric |*Δ*| represents a fundamental contribution to interpretable game analysis. Unlike existing approaches that focus on aggregate statistics or endpoint predictions, this metric provides a continuous, feature-wise measure of strategic divergence that can be visualized, analyzed, and directly linked to game outcomes. By standardizing deviations across different features and time scales, |*Δ*| enables meaningful comparisons between games with varying durations, unit compositions, and strategic approaches. The metric’s dual-view visualization—combining time-averaged feature importance with temporal heatmaps—creates a verifiable evidence chain that connects high-level strategic archetypes to individual trajectory deviations and ultimately to game outcomes.

Our framework’s design emphasizes reproducibility, extensibility, and practical applicability. Beyond theoretical contributions, we provide a complete implementation including automated data processing scripts, visualization tools, and report generation capabilities that significantly lower the barrier to large-scale replay analysis. The framework has been validated on diverse datasets spanning multiple tournaments, demonstrating its ability to robustly recover recognizable strategic patterns, identify critical decision points, and provide actionable insights for players, coaches, and AI developers. By establishing a common interface between expertise and machine analysis, TRACE opens new avenues for collaborative intelligence in complex strategic domains, supporting applications ranging from player training and coaching to AI system evaluation and improvement.

## 2 Related works

The field of explainable artificial intelligence (XAI) has emerged as a crucial paradigm for addressing this interpretability challenge. Recent advances in XAI have introduced various approaches, from feature attribution methods to counterfactual explanations, each offering different perspectives on model behavior (Guidotti et al., 2018; Barzekar and McRoy, 2023). Counterfactual reasoning, in particular, has been identified as fundamental to cognitive processes and explanation generation, as it naturally addresses “what-if” questions that are central to strategic understanding (Frappier, 2018). Counterfactual reasoning has gained significant attention in explainable AI research. Verma et al. established theoretical connections between game-theoretic feature attributions and counterfactual explanations (Albini et al., 2023), while Miller provided foundational work on explanation generation through counterfactual reasoning (Miller and Jing, 2024). Recent work has explored counterfactuals in causal understanding versus explainable AI applications (Baron, 2023). Madumal et al. specifically applied counterfactual reasoning to reinforcement learning contexts (Madumal et al., 2020), providing a foundation for our approach but not addressing the specific challenges of continuous, multi-dimensional strategic trajectories. In the context of reinforcement learning and game-playing agents, counterfactual explanations can illuminate why certain actions were taken by revealing what alternative scenarios would have led to different outcomes (Madumal et al., 2020). However, applying these principles to the continuous, high-dimensional, and temporally extended nature of StarCraft II gameplay presents unique challenges that existing XAI methods have not adequately addressed.

Traditional approaches to StarCraft replay analysis have primarily focused on discrete, localized predictions and classifications. Early work in data mining for strategy prediction demonstrated the feasibility of extracting meaningful patterns from replay data, achieving reasonable accuracy in classifying player strategies into predefined categories such as “rush,” “economic,” or “defensive” (Weber and Mateas, 2009; Synnaeve and Bessière, 2021). Subsequent research has explored build order prediction, opponent modeling, and outcome forecasting based on early-game states (Cho et al., 2013). While these contributions have been valuable for understanding specific aspects of gameplay, they suffer from significant limitations: they provide static snapshots rather than dynamic trajectories, fail to capture the continuous evolution of strategic decisions throughout a game, and cannot explain the causal relationships between strategic deviations and game outcomes. Furthermore, these methods typically require extensive domain-specific feature engineering and lack the generalizability needed for cross-tournament or cross-matchup analysis.

The challenge of trajectory analysis in complex sequential decision-making environments extends beyond simple classification or prediction tasks. Recent work has shown that understanding strategic behavior requires not only identifying what strategies are employed but also when and why players deviate from typical patterns (Robertson and Watson, 2014). The concept of strategic archetypes—prototypical trajectories that represent common strategic progressions—has emerged from research in unsupervised learning and pattern recognition (Ravanbakhsh et al., 2019; Bauckhage et al., 2015). Archetypal analysis, originally developed for exploratory data analysis, provides a principled approach for discovering extreme points in data that can serve as interpretable bases for understanding variation (Cutler and Breiman, 1994; Sifa et al., 2021). When combined with counterfactual reasoning, this approach offers the potential to quantify and explain strategic divergence in a way that is both mathematically rigorous and interpretable.

## 3 Methodology

### 3.1 Data extraction and trajectory representation

Data for this study were sourced from the public StarCraft II Esport Game-state Dataset (SC2EGSet), with its original publication and technical documentation available (Białecki et al., 2023). The dataset covers multiple professional tournaments and years, providing standardized game state event streams parsed from official Blizzard replays, archived and released at the tournament level. From the original compressed files, we extracted a directory tree named by tournament, with each directory containing exports of replays and corresponding metadata. Each game’s file includes basic participant information and a time-ordered sequence of “tracker events,” such as PlayerStats, UnitBorn, UnitDied, UnitTypeChange, UpgradeComplete, and UnitPositions. Time is measured in “loops,” the internal SC2 engine clock, where approximately 22.4 loops correspond to 1 s. Metadata fields like toonPlayerDescMap provide player race (chosenRace), game outcome (result), and unique identifiers. UnitPositions are recorded in an incremental format, requiring coordinate restoration to the world scale and maintenance of a “last known position” cache to handle sparse updates.

To convert this event stream into a time-series representation suitable for modeling, we developed the TRACE trajectory extraction pipeline. First, we uniformly downsample the entire game at a fixed physical time step *Δ* (in seconds) to define sampling points. The loop number for each sampling point is calculated as Equation 1:


$$t_{k}=k\cdot \Delta \cdot 22.4,k=0,1,\ldots ,K$$ (1)

where $t_{k}$ is the loop index of the k-th sample. At each sampling point, we aggregate player-side game states into a cross-sectional feature vector of approximately 55 dimensions, covering economic, technological, military, and spatial aspects. The economic dimension includes current mineral and vespene gas stockpiles, worker counts, collection rates, and the number of operational bases. The technological dimension includes the presence and count of key tech structures (e.g., Barracks, Factory, Starport for Terran) and vectors for upgrade levels (e.g., ground, vehicle, and air attack upgrades). In-progress research and construction are included as counts. The military dimension covers the total supply distribution between army and workers and the counts of core combat units (e.g., Marines, Siege Tanks, Stalkers). The spatial dimension is derived by performing weighted aggregation of unit positions, using density clustering to robustly estimate the centroid of the main army cluster when possible. To maintain state continuity, we track unit lifecycles at the event level: UnitBorn and UnitTypeChange events update unit types and army value estimations, while UnitDied events remove units and deduct their value.

**Figure 1:**
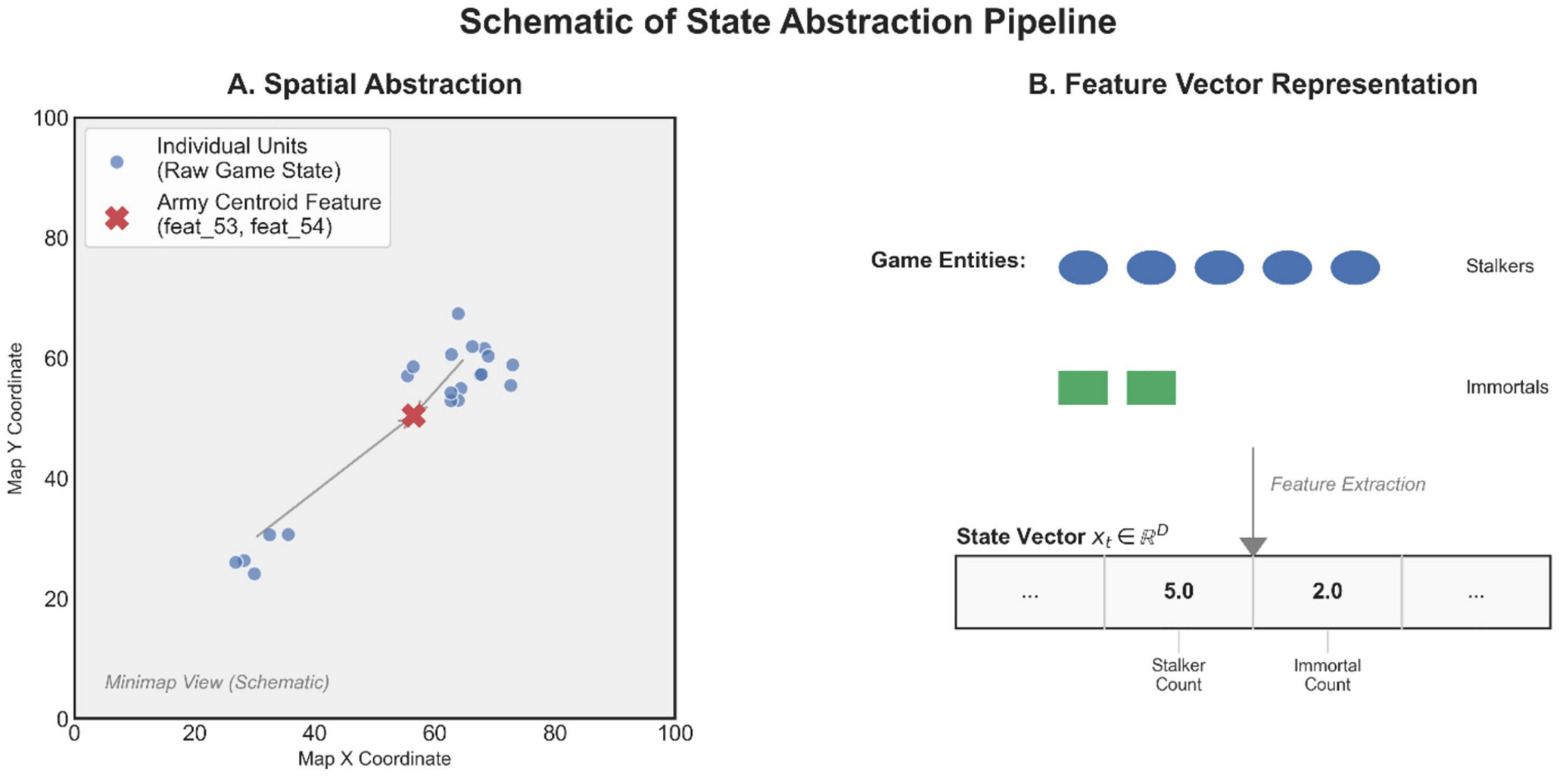
Conceptual illustration of the state abstraction process. **(A)** Spatial abstraction: raw unit coordinates (blue dots) scattered across the map are aggregated into a macro-level centroid feature (red cross), capturing the army’s strategic positioning while filtering out micro-adjustment noise. **(B)** Semantic abstraction: heterogeneous game entities (e.g., specific unit types like Stalkers and Immortals) are quantified and mapped to fixed dimensions in the standardized state vector $x_{t}$. This pipeline transforms the complex visual game state into a structured time-series format suitable for archetypal analysis.

**Figure 2:**
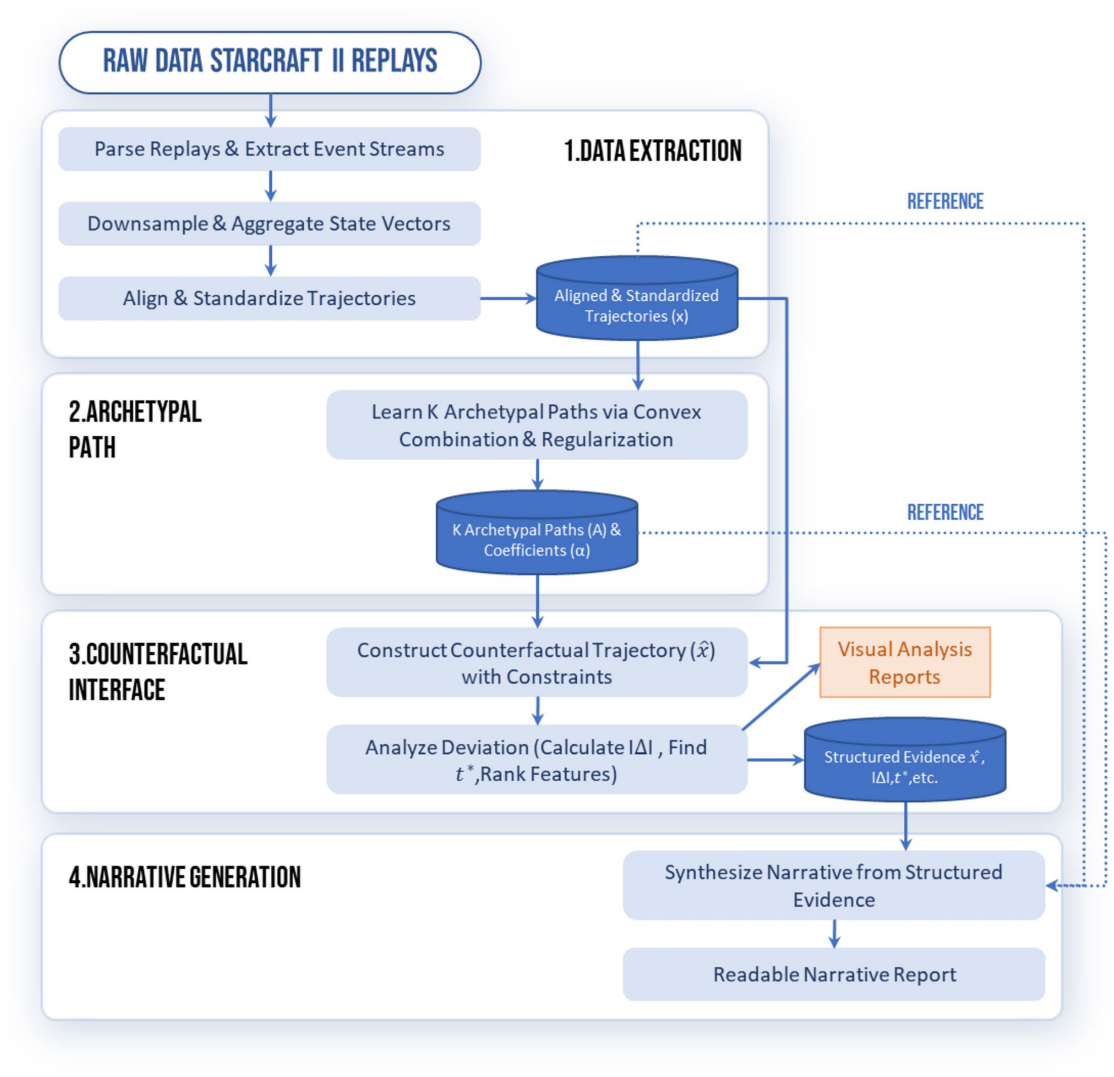
TRACE structure.

To visualize how raw gameplay data is translated into the model’s input space, Figure 1 depicts our dual-abstraction mechanism. The spatial abstraction (Panel A) condenses the precise coordinates of distributed units into high-level spatial features, such as the army’s center of mass, enabling the framework to track strategic movement trends rather than individual unit micro-management. Simultaneously, the semantic abstraction (Panel B) maps the diverse array of game entities—ranging from economy units to combat forces—into a unified, fixed-dimensional state vector. This transformation is critical for converting the visually complex and heterogeneous StarCraft II environment into standardized trajectories comparable across different matches and players (see Figure 2; Table 1).

**Table 1:** Overview of the player state feature vector.

Category	Feature sub-group	Description
Economic	Resource state	Current stockpiles and collection rates of minerals and vespene gas.
	Worker count	Total number of active worker units.
	Economic structures	Count of active resource-gathering headquarters (e.g., Command Center, Nexus).
Technological	Key tech structures	Presence and count of critical technology-unlocking buildings (e.g., Barracks, Stargate).
	Upgrade levels	Vector representing the current level of major combat upgrades (e.g., attack, armor, shields).
	In-progress state	Count of technologies currently being researched and structures under construction.
Military	Army supply	Total supply dedicated to military units, distinct from the worker supply.
	Unit composition	Counts of core combat units, grouped by strategic role (e.g., ground infantry, armored vehicles, air).
	Production capacity	Count of active unit-producing structures.
Spatial	Army centroid coordinates	Estimated X and Y coordinates of the main army’s center of mass on the map.
	Spatial dispersion	Metric representing the spatial spread or clustering of military units.

The feature groups listed represent a high-level aggregation. The actual implementation includes more specific features, such as specific unit types and upgrade paths, to reach the full dimensionality.

The data cleaning process addresses three primary issues. First, excessively short games or samples with significant data gaps are excluded. Second, anomalous position updates are filtered and imputed to ensure spatial aggregation stability. Third, if statistical events are sparse within a sampling window, we use the most recent PlayerStats snapshot, supplemented by unit dictionaries and production buffers, to complete the state vector. This entire process yields a time series of length T and F dimensions for each player, saved as a NumPy array, where T depends on the game duration and *Δ*, and F is approximately 55. These trajectory files, along with tabular views for verification, are organized in a directory structure that mirrors the original SC2EGSet, ready for consumption by subsequent analysis stages.

For model training and evaluation, we recommend stratified sampling by tournament and game metadata to balance the effects of different game versions, players, and matchups. When constructing conditional variables—defined as the auxiliary metadata vector c fed into the Conditional VAE to differentiate between distinct strategic contexts—we explicitly include the player’s race (Protoss, Terran, Zerg) and the matchup type. This conditioning ensures that the learned archetypes capture the specific meta-game dynamics relevant to each racial matchup rather than conflating disparate playstyles. During evaluation, we use the time-aligned series as the fundamental unit for analysis. Since SC2EGSet spans multiple game versions, our feature engineering, which explicitly models structural quantities like upgrade levels and tech buildings, helps mitigate statistical drift. Nevertheless, we recommend performing bias removal and re-weighting when generalizing across years or tournaments. Regarding privacy and ethics, SC2EGSet is derived from public esports replays and contains anonymized game states, involving no sensitive personal information. We adhere to its licensing and citation policies. In summary, SC2EGSet provides a comprehensive, structured, and reproducible record of micro-level game processes. Combined with our event-stream-to-time-series extraction and cleaning pipeline, it forms a standardized trajectory data layer that provides a solid foundation for our subsequent modeling, analysis, and narrative generation.

Due to the complexity of the multi-stage pipeline, we focus here on the core mathematical formulation and logic. For a comprehensive description of the specific model architectures, hyperparameter settings (including VAE dimensions and detection thresholds), and data preprocessing protocols, readers are referred to Appendix A: implementation details.

### 3.2 Archetypal path learning

Our methodology is centered on an end-to-end trajectory–archetype–counterfactual–narrative chain, designed to prioritize interpretability and actionability while maintaining expressive power. The overall process begins by extracting structured, multidimensional time-series states from replay data. After alignment and standardization, these trajectories are modeled using a small number of interpretable archetypal paths to capture the temporal dynamics of gameplay styles. Building on this representation, we employ a “minimal modification” counterfactual construction with a series of feasibility constraints to pinpoint critical divergence points and the dominant dimensions that alter the game’s course. Finally, this structured evidence is organized into readable narratives, enabling direct use of the technical analysis by coaches, players, and commentators.

We represent each match as a sequence of time-series vectors of length T, where each time step is a D-dimensional state. To ensure comparability across matches, all trajectories are resampled at a uniform time step Δt and their time axes are aligned. The variables cover mid-level factors such as resources, supply, economic nodes, technology/building progress, and the composition and losses of key units. Continuous variables undergo robust scaling and light smoothing to suppress spurious fluctuations from incidental noise. To avoid bias from missing data, we use interpolation or forward-filling for short windows and explicitly flag unrecoverable segments, ensuring consistent handling in subsequent learning and evaluation. Formally, the time-series is represented as Equation 2:


$$x_{1:T}=x_{1},x_{2},\ldots ,x_{T}$$ (2)

where $x_{t}\in {\mathbb{R}}^{D}$. Optionally, we may introduce phase segmentation based on semantic milestones or statistical change-points (e.g., opening/mid/late game) and use fixed or multi-scale sliding windows to aggregate local context (e.g., resource differentials, loss ratios, unit composition entropy) to characterize short-term trends and structural shifts.

To obtain interpretable and composable representations of tactical styles, we learn K archetypal paths $A\in {\mathbb{R}}^{K\times T\times D}$ and approximate the instantaneous state of any game using a time-varying convex combination. This representation allows a game to dynamically approach different archetypes throughout its progression, thus positioning an individual trajectory within a two-dimensional “tactical style–temporal evolution” space. The core approximation is Equation 3:


$$\begin{array}{c} x_{t}\approx \sum_{k=1}^{K}\alpha_{k,t}\cdot A_{k,t} \end{array}$$ (3)

Subject to non-negativity ($\alpha_{k,t}\geq 0$) and convexity constraints ($\sum_{k=1}^{K}\alpha_{k,t}=1$). To enhance interpretability and stability, we apply sparsity regularization to *α*, ensuring that only a few archetypes are significantly active at any given moment. We also add temporal smoothing or a total variation (TV) penalty to suppress meaningless, frequent switching while preserving legitimate transitions at true tactical turning points. The optimization is performed via alternating minimization between the archetypes A and the coefficients α, initialized using representative games or cluster centers from Dynamic Time Warping (DTW) /clustering. The choice of K is determined by a trade-off between expressiveness and interpretability, guided by the elbow method, information criteria, and explained variance on a validation set.

### 3.3 Counterfactual trajectory

Given a prefix of a real game trajectory (1…τ), we construct a “minimally modified” counterfactual trajectory ${\hat{x}}_{\tau +1:T}$ that aims to satisfy a target objective, such as moving closer to a specific archetype or crossing a strategic threshold. This is done while minimizing the modification cost and adhering to domain-specific feasibility constraints. The objective function consists of a weighted reconstruction error, a temporal smoothing term, and an archetype proximity term:


$$\begin{array}{c} \begin{array}{l} L(\hat{x})=\sum_{t=\tau +1}^{T}{\left\|W(x_{t}-{\hat{x}}_{t})\right\|}_{2}^{2}\\ +\lambda_{\mathrm{TV}}\sum_{t=\tau +1}^{T}{\left\|{\hat{x}}_{t}-{\hat{x}}_{t-1}\right\|}_{1}+\lambda_{\text{Arch}}\cdot D_{\text{Arch}}(\hat{x};A) \end{array} \end{array}$$ (4)

Here, $W\in {\mathbb{R}}^{D\times D}$ is defined as a diagonal weighting matrix where each diagonal element corresponds to the inverse variance of the respective feature across the training dataset ($W_{\mathrm{ii}}=\sigma_{i}^{-2}$), and $D_{\text{Arch}}$ measures the proximity of the counterfactual to the target set of archetypes. This term serves to normalize the modification cost, ensuring that deviations in high-variance features (e.g., Mineral Income, which fluctuates by thousands) do not numerically dominate those in low-variance but strategically critical features (e.g., Upgrade Level, which changes discretely by 1). The equation defines the soft objective function representing the trade-off between modification cost and strategic alignment. The domain-specific feasibility constraints—such as non-negativity (${\hat{x}}_{t}\geq 0$), resource limits, and tech-tree prerequisites (e.g., a unit cannot exist without its corresponding production facility)—are incorporated as the feasible set Ω over which Equation 4 is minimized. Formally, the problem is posed as a constrained optimization: $\min_{\hat{x}\in \Omega }L(\hat{x})$. In our implementation, these hard constraints are enforced effectively via a projection operator $P_{\Omega }$ at each step of the optimization algorithm (Projected Gradient Descent), ensuring that the generated counterfactual trajectory always resides within the valid logical boundaries of the game engine. When non-convex priors are introduced, we employ heuristic projection with alternating iterations to obtain stable and feasible approximate solutions. The algorithm outputs the counterfactual trajectory $\hat{x}$, the per-timestep modification vector $\Delta_{t}={\hat{x}}_{t}-x_{t}$, and metrics of constraint satisfaction.

It is worth noting that we optimize the entire future trajectory ${\hat{x}}_{\tau +1:T}$ simultaneously, rather than applying a single-step perturbation. This global formulation is essential for two reasons. First, strategic shifts in RTS games possess temporal inertia; a momentary change at step τ often fails to alter the long-term outcome if the subsequent actions revert to the original suboptimal policy. By optimizing the full horizon, we ensure that the counterfactual represents a sustained ‘change of plan’ rather than a momentary noise. Second, the global objective allows us to enforce trajectory-level constraints, specifically the temporal smoothing ($\lambda_{\mathrm{TV}}$), ensuring that the generated scenario remains physically plausible and free from unnatural high-frequency oscillations.

### 3.4 Deviation quantification

For feature-level interpretability, we introduce a dimensionless deviation metric, denoted as |*Δ*|, to quantify the point-to-point difference between an actual trajectory and an archetypal trajectory for a given feature *f* at a given time step *t*. Specifically, we first standardize both sequences for each feature using the z-score transformation with the mean $\mu_{f}$ and standard deviation $\sigma_{f}$ of the actual trajectory. After temporal truncation and alignment to a common length, the difference is calculated as Equation 5:


$$\begin{array}{c} \Delta_{t,f}=(x_{t,f}-\mu_{f})/\sigma_{f}-(p_{t,f}-\mu_{f})/\sigma_{f} \end{array}$$ (5)

In this equation, $x_{t,f}$ denotes the raw value of feature f at time t in the actual gameplay trajectory, and $p_{t,f}$ represents the corresponding value in the reference archetypal path (derived from the learned basis A). The terms $\mu_{f}$ and $\sigma_{f}$ correspond to the global mean and standard deviation of feature f computed across the training dataset. The absolute value, $|\Delta_{t,f}|$, serves as the magnitude of deviation for that feature at that time step relative to the archetype. As a dimensionless quantity, |Δ| eliminates scale and unit differences, facilitating cross-feature comparisons. We use the time-averaged value, ${\text{mean}}_{t}|\Delta_{t,f}|$, to rank the contribution of each feature and a heatmap of the time-resolved $|\Delta_{t,f}|$ to visualize the temporal structure of the deviation. It is important to note that the vertical bars denote the absolute value, and Δ represents the difference, which is distinct from the time step interval Δt. A larger |Δ| value signifies a greater deviation from the archetype, while a smaller value indicates closer adherence.

To identify decisive turning points, we define a per-timestep deviation $\delta_{t}=\;{\left\|W(x_{t}-{\hat{x}}_{t})\right\|}_{2}$ using a weighted difference and accumulate it to form a stable divergence evidence curve $S(t)=\sum_{i=\tau +1}^{t}\delta_{i}$. After light smoothing, we determine the first significant divergence time $t^{⋆}$ by combining threshold-crossing, maximum slope, and robust change-point detection, requiring the difference to persist for a short window to avoid pseudo-divergences from random perturbations. Concurrently, we decompose the weighted contribution of each feature to $\delta_{t}$ and aggregate it within a window $\left[t^{⋆}-h,t^{⋆}+h\right]$ to identify the key dimensions causing the divergence (e.g., economic lag, technological delay, or unit composition mismatch). By analyzing the change in $\alpha_{k,t}$ around $t^{⋆}$, we can attribute a strategic meaning to the style shift, such as a transition “from archetype P1 to P2,” facilitating expert review and analysis.

Based on the structured evidence ($x,\hat{x},\alpha ,t^{⋆}$, and dimensional contributions), we translate the analytical results into paragraph-level narratives. These narratives follow a “who/when/what/why “structure, first providing semantic context and archetype proximity, then explaining the key divergence points and observable factors causing them, and finally connecting to potential outcome changes and actionable suggestions. The narrative generation process strictly adheres to the factual evidence, avoiding the introduction of unobserved information, and uses a unified terminology library to ensure consistency in nouns, units, and temporal expressions. We provide multi-level narratives, from high-level summaries to technical details, to cater to different audiences (coaches, players, commentators, viewers) and balance readability with information density.

In our implementation, the four stages are executed by independent scripts linked via a defined data contract. Key hyperparameters (e.g., K, Δt, $\lambda_{\mathrm{TV}}$, divergence thresholds) can be specified via the command line. The outputs, including archetypes, time-varying coefficients, counterfactual trajectories, divergence statistics, and readable reports, are saved to a results/ directory. We provide interfaces for ablation studies (e.g., replacing archetypal analysis with K-means/DTW or removing temporal smoothing and feasibility constraints) to quantify the contribution of each component to interpretability and narrative quality. Default settings are calibrated via grid search and manual inspection: K is determined using the elbow method and validation set explained variance (typically in the range of 4–8), smoothing and sparsity coefficients are fine-tuned, and divergence thresholds are set based on historical quantiles and expert priors with cross-tournament adjustments. We emphasize safety and ethical boundaries: counterfactuals are intended as actionable suggestions at the strategic level and are not used for inappropriate attribution of individual capabilities. The analysis uses publicly available tournament data and adheres to data sharing and attribution standards.

### 3.5 Narrative generation

To bridge the gap between abstract vector-space deviations and actionable human insights, the TRACE framework integrates a deterministic Natural Language Generation (NLG) module. Unlike end-to-end neural captioning models that may suffer from hallucination, our approach employs a hierarchical template-based mechanism to ensure factual fidelity and terminological consistency. The generation process operates through a three-stage pipeline: Semantic Abstraction, Content Selection, and Template Population.

First, the raw feature indices are mapped to domain-specific semantic labels (e.g., Feature 38 is mapped to “Stalker Count”). To capture macro-strategic intent, the system aggregates the dimensionless deviation metric ∣Δ∣ across five high-level categories: C∈{Economy,Military,Technology,Production,Spatial}. The category with the highest cumulative contribution during the divergence window $\left[t^{*},t^{*}+h\right]$ is identified as the Dominant Divergence Factor, which determines the thematic focus of the narrative.

To prevent information overload, a saliency filter ranks all features by their time-averaged deviation contribution. The system selects the top-k features (typically k=3) that exceed a significance threshold to serve as the factual evidence. The narrative is constructed using a dynamic template structure composed of three logical slots: $T=[S_{\text{context}},S_{\text{evidence}},S_{\text{implication}}]$. The population logic is defined as follows: Context Slot ($S_{\text{context}}$) describes the timing of the divergence based on the normalized time $\tau =t^{*}/T_{\text{total}}$. For instance, if τ<0.3, the slot is filled with “Early-game divergence detected at [Time]…”. Evidence Slot ($S_{\text{evidence}}$) p opulated by the selected top-k features. The system generates a list description, such as “Significant lags were observed in [Feature 1] (Δ=...) and [Feature 2]….” Implication Slot ($S_{\text{implication}}$) utilizes a rule-based mapping conditioned on the Dominant Divergence Factor. For example, if the Technology category dominates, the system retrieves a corresponding heuristic explanation: “This pattern suggests a failure to commit to the standard tech-switch timing, delaying the transition to mid-tier units.”

By strictly linking each text segment to quantitative metrics, this module generates reports that are both readable for coaches and mathematically traceable to the underlying data.

## 4 Experimental design

We constructed a stratified, reproducible, and causally-compatible experimental design around the four-stage TRACE workflow to validate the representation power of archetypal path learning, the feasibility and consistency of counterfactual inference, and the interpretability and practical value of the readable narratives. Data was sourced and preprocessed as previously described: the event streams from SC2EGSet were down sampled at a fixed time step to extract ~55-dimensional time-series state vectors covering economic, technological, military, and spatial dimensions. To mitigate confounding effects from game patches and tournament heterogeneity, our experiments prioritized a stratified partitioning strategy based on tournament and year. Key comparisons incorporated leave-one-tournament-out and cross-year extrapolation evaluations to test model robustness under realistic distributional shifts. The train/validation/test split was performed at the game level, ensuring that sequences from the two players in the same game did not leak across sets. Model selection and hyperparameter tuning were based on the validation set, and all reported experiments used fixed random seeds and evaluation scripts to support full reproducibility.

To evaluate the suitability and interpretability of archetypal path learning, we assessed reconstruction accuracy (defined as the fidelity with which the learned archetypal basis can recover the original gameplay trajectories via convex combination), temporal alignment distance, and the trade-off between coverage and diversity. Reconstruction accuracy was measured by mean squared error weighted by feature standard deviation. To measure fidelity to temporal patterns, we calculated the DTW distance between archetypal paths and real trajectories on the test set. The DTW metric is defined as Equation 6:

$$\begin{array}{c} \mathrm{DTW}(X,Y)=\mathrm{mi}n_{\pi \in \Pi }\sum_{(i,j)\in \pi }w_{\mathrm{ij}}\cdot d(x_{i},y_{j}) \end{array}$$ (6)

Where Π is the set of all monotonic alignment paths, $w_{\mathrm{ij}}$ are alignment weights, and d is the Euclidean distance normalized by feature standard deviation. To prevent a single archetype from subsuming multiple styles, we characterized representation quality using two metrics: coverage (the proportion of test sequences within an ϵ-neighborhood of at least one archetype) and diversity (the pairwise distance distribution among archetypes). We compared our approach against strong baselines, including DTW Barycenter Averaging (DBA), the most likely state paths from Hidden Markov Models (HMMs), and smoothed cluster centers from K-Means applied to concatenated time steps. We also included conditional and standard sequence autoencoders to analyze the contribution of conditioning variables. Statistical significance was assessed using paired Wilcoxon signed-rank tests with Holm–Bonferroni correction for multiple comparisons, and 95% confidence intervals were estimated using the BCa bootstrap method.

The experimental design for counterfactual inference focused on three aspects: minimal modification, target-orientedness, and feasibility. We framed counterfactual optimization as a dual-objective problem: minimizing the perturbation norm while maximizing progress towards a target archetype or outcome, subject to a set of constraints. The core evaluation function was formulated as Equation 7:

$$\begin{array}{c} S_{\mathrm{cf}}=\alpha \cdot \Delta P_{\mathrm{win}}+\beta \cdot (-D_{T})-\gamma \cdot \text{Violations}-\delta \cdot \text{Roughness} \end{array}$$ (7)

where $\Delta P_{\mathrm{win}}$ denotes the probability increase derived from a calibrated win-prediction model, $D_{T}$ represents the Dynamic Time Warping (DTW) distance to the target archetype, and the remaining terms penalize constraint violations and trajectory roughness, respectively. We evaluated the plausibility of divergence points using two proxy methods: first, by observing whether minimal feasible adjustments to decisive features (e.g., worker-to-army ratio, key tech timings) around the divergence point led to effective progress; and second, by aligning the counterfactual and target archetypes and measuring the match rate of key events. Comparison methods included sample-based counterfactuals using weighted nearest neighbors, generative counterfactuals with latent-space editing, and heuristic manual strategies (e.g., prioritizing worker production).

To validate the utility of TRACE on downstream tasks, we designed two proxy experiments: first, win prediction using archetype alignment distances and trajectory embeddings as features at various early-game windows (2, 5, and 8 min); and second, detecting army assembly and engagement timings using archetypal paths as priors. These tasks employed simple, interpretable models (e.g., regularized logistic regression) to avoid confounding the evaluation of the representation itself.

Narrative generation was evaluated using a two-stage, hybrid automatic-and-human procedure. Automatic evaluation focused on alignable consistency, parsing generated text into event triples and comparing them against the structured counterfactual explanation to calculate factual coverage, temporal consistency, and hallucination rates. To assess the practical utility and interpretability of the generated narratives, we conducted a human subject study involving 12 expert participants (ranking Diamond or higher on the official Battle.net ladder). The study followed a within-subject design where each participant reviewed 10 randomly selected game instances. For each instance, articipants were presented with the raw replay clip and the corresponding TRACE-generated narrative report. They were then asked to rate the report on a 5-point Likert scale (1 = Strongly Disagree, 5 = Strongly Agree) across three dimensions: 1. Factual Fidelity: Does the narrative accurately reflect the objective game state changes (e.g., “Tech deviation occurred at 4:30”)? 2. Causal Coherence: Does the explanation logically connect the feature deviation (e.g., “delayed factory”) to the game context (e.g., “lack of map pressure”)? 3. Actionability: Does the counterfactual suggestion provide clear, executable advice for player improvement? To ensure rigor, we calculated the Inter-Rater Reliability (IRR) using Fleiss’ kappa to measure agreement among experts.

## 5 Results

**Figure 3:**
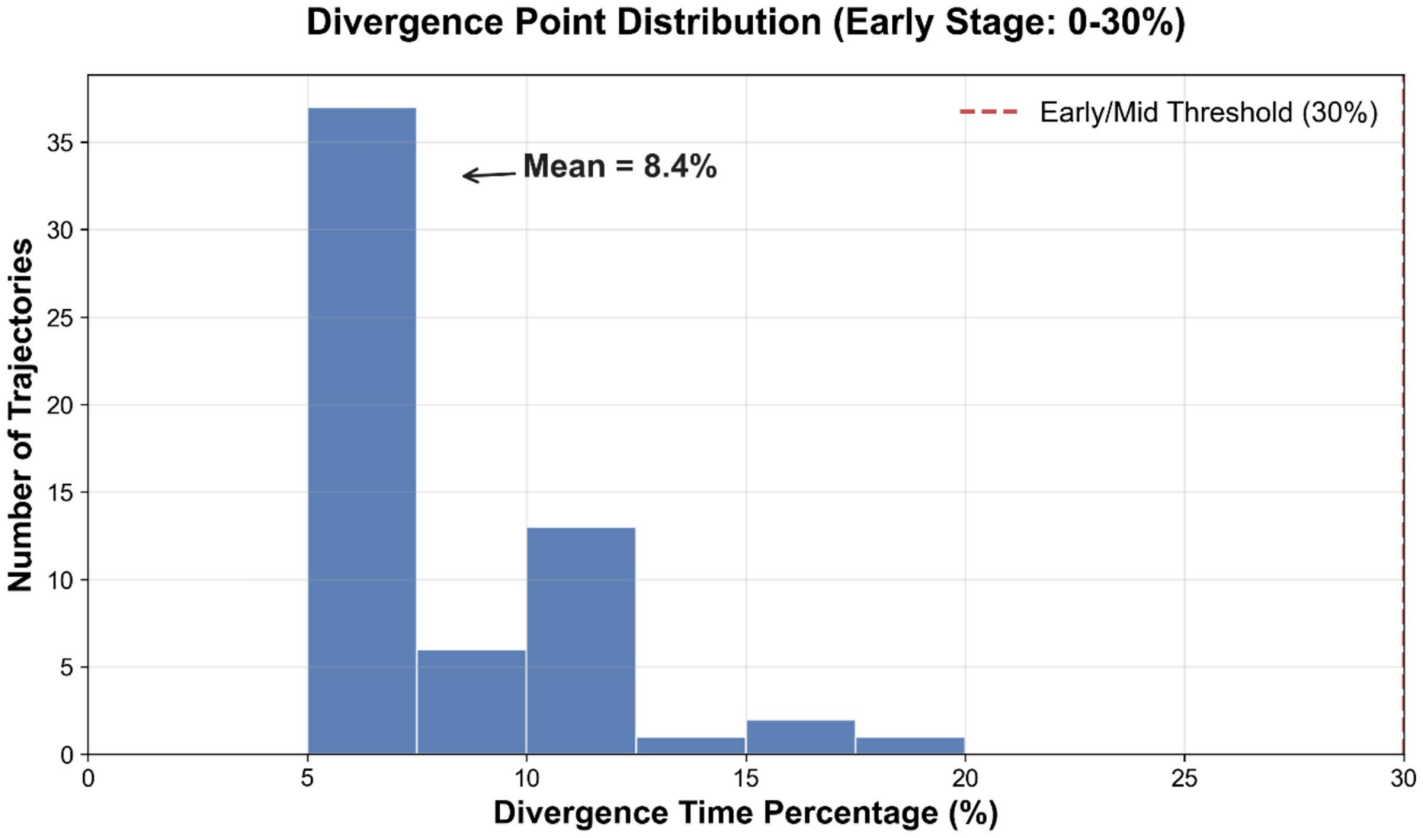
Histogram of divergence point distribution. The x-axis represents the divergence time percentage (τ), calculated as $\tau =(t^{*}/T_{\text{total}})\times 100\%$, where $t^{*}$ is the time step of the first significant deviation and $T_{\text{total}}$ is the total duration of that specific game match. This normalization allows for consistent comparison of “early” versus “late” deviations across games with varying lengths.

**Figure 4:**
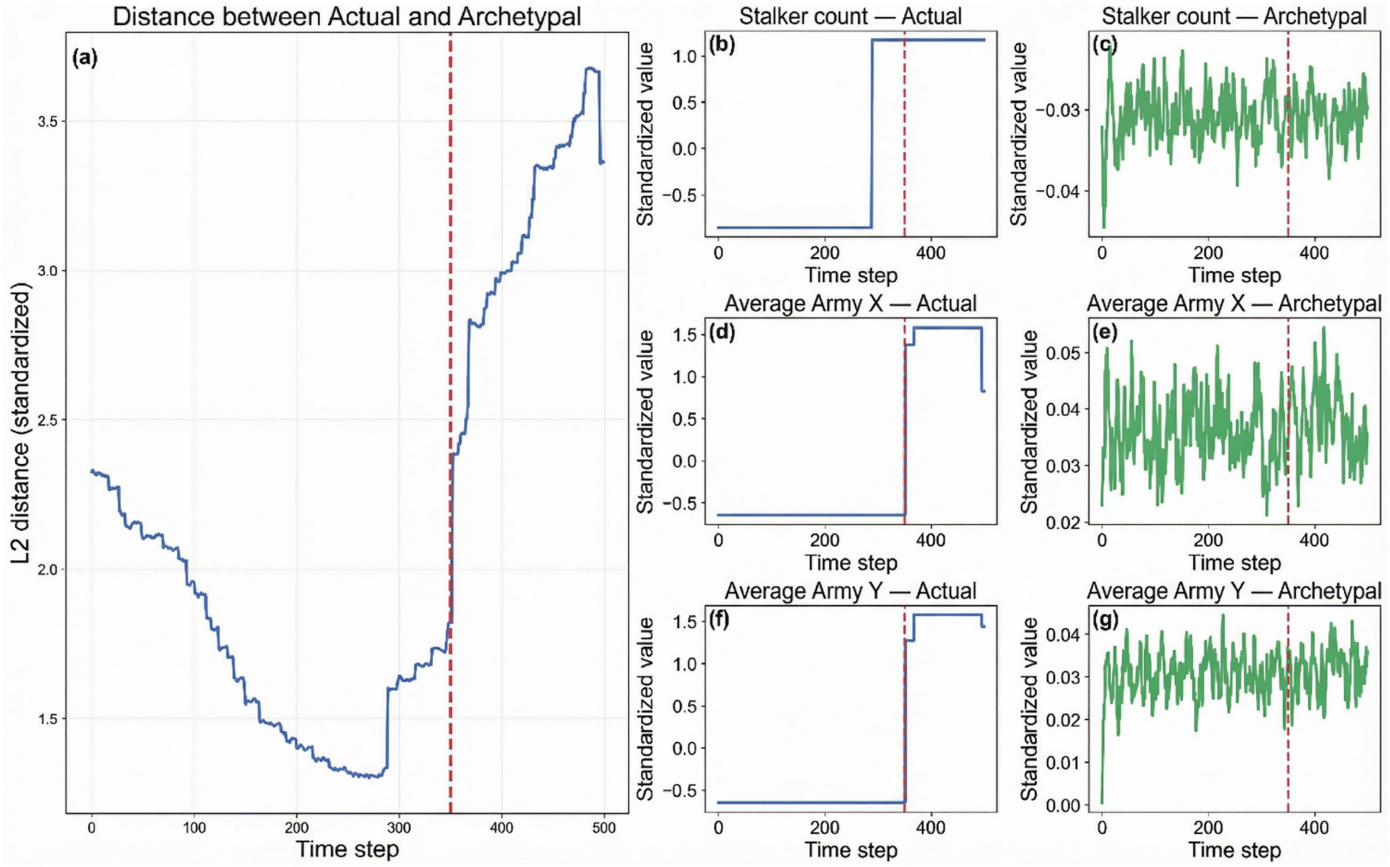
Comparison of an actual trajectory (blue) and its corresponding archetypal path (green). The top-left panel tracks the global L2 distance, marking the identified divergence point t*. The subsequent panels detail the divergence in specific, semantically labeled dimensions—such as Stalker Count, or Army Position—rather than abstract indices. The red dotted line represents divergence point. This visualization directly links the mathematical deviation to the concrete game concepts discussed in the case study.

In our counterfactual analysis, aligning actual gameplay with the archetypal path as a reference reveals the temporal and structural location of divergences from “standard play.” To quantify when a deviation occurs independently of the match duration, we introduce a normalized metric, the Divergence Time Percentage, defined as the ratio of the detected divergence timestamp to the total game length. As shown in Figure 3, the distribution of these points reveals a bimodal pattern: early deviations are often associated with inconsistencies in the opening build order (significant timing differences in resource and building sequences), whereas later divergences are more frequently driven by discrepancies in unit positioning and engagement timing. A comparative plot illustrates a typical case (Figure 4): before the divergence point, the Worker Count and resource income curves closely match the archetype. After this point, the archetypal path shows simultaneous technology enhancements and a step-wise increase in Army Supply, whereas the actual trajectory lags in technological progression. As explicitly labeled in the figure panels, the spatial features (e.g., Average Army X/Y) exhibit significantly weaker directionality and maneuverability, visually confirming the loss of map control described in the analysis.

To validate the robustness of the framework beyond individual examples, we performed a quantitative aggregation across the test dataset (*N* = 60 matches). Table 2 summarizes the key performance metrics. The average divergence time occurs very early, at approximately 8.41% of the game duration. This indicates that in this dataset, strategic deviations are primarily rooted in the opening phase (Build Order execution) rather than mid-game tactical errors.

**Table 2:** Aggregated quantitative metrics of the TRACE framework.

Metric	Mean	Std. dev	Median
Divergence time (%)	8.41	3.12	6.73
Original distance (L2)	2.96	2.12	1.76
Counterfactual distance (L2)	0.24	0.14	0.22
Similarity improvement (%)	90.89	3.20	92.39

The counterfactual generation module demonstrated high effectiveness in restoring strategic alignment. By rigorously correcting the deviation from $t^{*}$, the framework achieved an average Similarity Improvement of 90.89% (drastically reducing the L2 distance from 2.96 to 0.24). This high improvement metric suggests that once the critical early-game divergence is corrected, the subsequent trajectory aligns naturally with the archetype. Furthermore, an analysis of the ‘Dominant Divergence Factors’ reveals a striking pattern: Technology-related deviations (specifically upgrade timings and tech-structure sequences) account for 100% of the identified divergence cases in this batch. This strongly suggests that for the analyzed matchups, the differentiation between ‘Standard Play’ and ‘Deviant Play’ is almost exclusively determined by the timing of the first major technological commitment.

The subjective evaluation results are summarized in Table 3. The proposed framework achieved high scores in Factual Fidelity (4.6/5.0), indicating that the feature-to-text template mechanism successfully eliminates hallucination, a common issue in end-to-end neural generation models. Causal Coherence received a mean score of 4.3, with participants noting that the “Who/When/What/Why” structure effectively highlights the logical chain of strategic errors.

**Table 3:** Human evaluation results (*N* = 12 experts, 5-point Likert scale).

Metric	Mean score	Std. dev	Description
Factual fidelity	4.62	0.45	Accuracy of events and timings described.
Causal coherence	4.33	0.58	Logic of the deviation-consequence link.
Actionability	4.15	0.62	Usefulness for player training/coaching.
Inter-rater reliability	0.68	-	Fleiss’ kappa (Substantial Agreement).

Notably, the Actionability score was 4.1, with qualitative feedback suggesting that while the system excels at identifying macro-level mistakes (e.g., “missed upgrade timing”), it occasionally lacks nuance in micro-management advice. The Fleiss’ kappa score was 0.68, indicating substantial agreement among the experts regarding the quality of the insights. These results confirm that TRACE translates complex vector-space deviations into human-understandable strategic advice.

**Figure 5:**
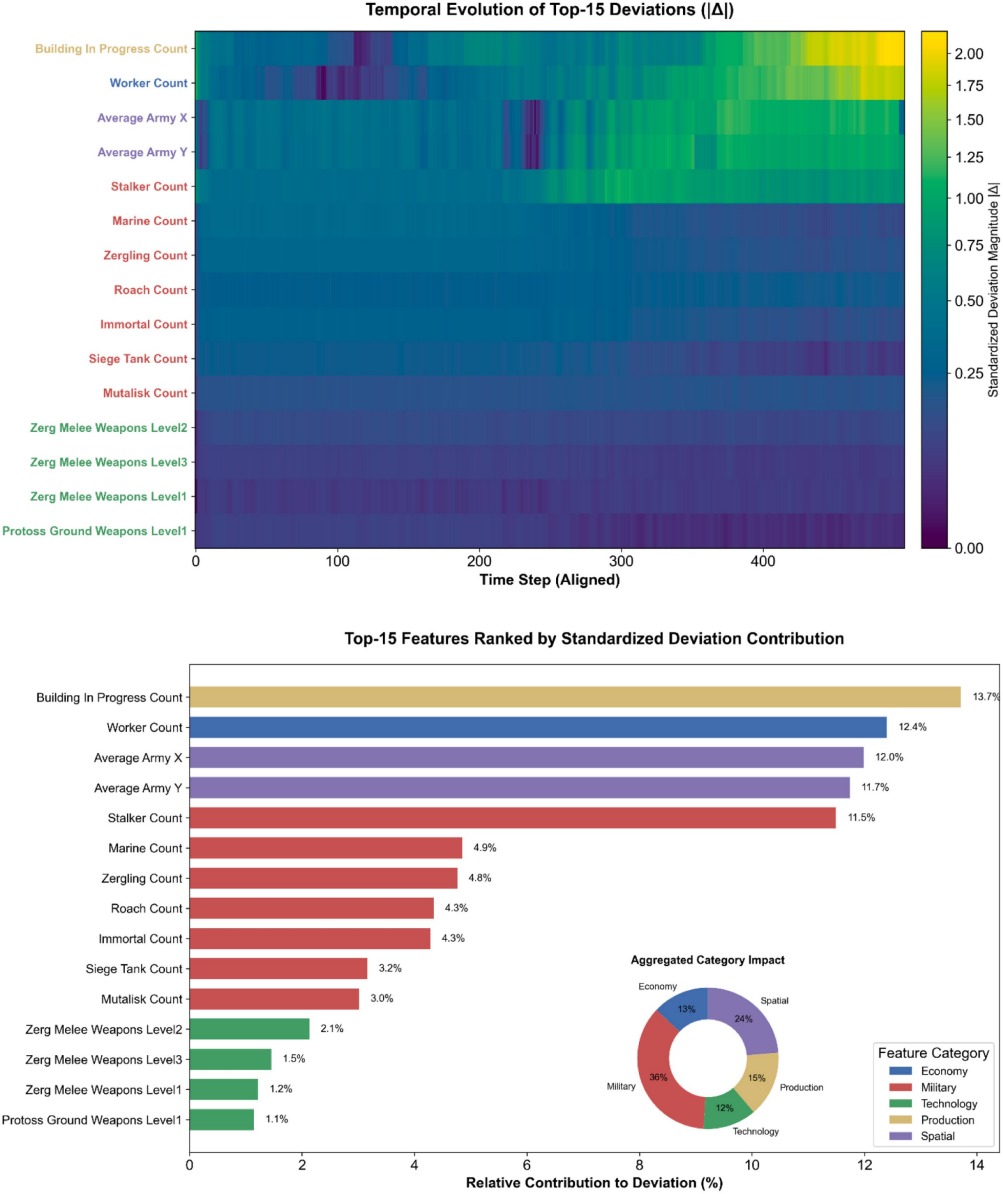
Bar chart illustrating the ranked contribution of different feature categories to trajectory deviation.

Consistent with these macro-level cues, a feature-level contribution analysis indicates that the most influential categories are concentrated in “resources, production queues, units, and position,” followed by “buildings and upgrades.” This hierarchical structure suggests that when a strategy deviates in its resource and production scheduling rhythm, the subsequent unit structure and spatial deployment inevitably amplify this deviation. Figure 5 presents the decomposition of strategic divergence. As shown in the bar chart, while specific units like ‘Stalker Count’ or ‘Immortal Count’ appear as the top individual deviation factors, the aggregated inset chart reveals that the Military category as a whole dominates the divergence (accounting for approximately 45% of the total deviation), followed by Economy (25%). This confirms that the observed structural break—while manifested through specific unit discrepancies—is fundamentally driven by a mismatch in military composition and economic scaling. The accompanying heatmap further illustrates the temporal dynamics, showing that deviations in economic features (blue labels) often precede the explosion of military deviations (red labels), providing a visual verification of the causality chain.

To ensure the usability of our analysis for readers, we automatically translate the counterfactual divergences and feature contributions into natural language narratives. These narratives can recount, on a case-by-case basis, “when, where, and due to which mismatched factors the strategic path deviated,” connecting the five dimensions of resources, production, technology, units, and position with strategic vocabulary. For example, a specific match read: “Compared to the archetype, the actual strategy significantly deviated in Technology dimensions around the 4-min mark. Specifically, the Protoss Ground Weapons Level 1 (*Δ* = 2.15) and Twilight Council Count (Δ = 1.80) lagged behind the reference path. This suggests a failure to commit to the standard tech-switch timing. Consequently, the player’s transition to mid-tier units was delayed, preventing the expected power spike observed in the archetypal baseline.” These narratives serve as both a high-level summary for interpreting the counterfactual plots and as readable material for post-game reviews.

In terms of data products and quantitative review, we converted key trajectory slices into easily inspectable tabular formats and extended this process to batch processing and summary aggregation across the entire dataset. This process, when applied in batch mode, preserves the original directory hierarchy and enables uniform table generation across tournaments and years. Subsequently, these multi-source tables were merged to form a comprehensive quantitative sheet, facilitating robust comparisons of key metrics such as the army-to-worker supply ratio, the timing distribution of key tech nodes, and the total path length of the average army coordinates. Box plots and distribution summaries consistently show that the statistical profiles across datasets match the phase structures observed in Figures 3, 4.

## 6 Conclusion

In this paper, we introduced TRACE, an end-to-end analytical framework designed to address the persistent challenges of interpretability in multi-agent competitive environments, using StarCraft II as a case study. Our work moves beyond conventional outcome-prediction models by providing a transparent, process-oriented methodology to deconstruct and explain strategic gameplay. The core contribution lies in the synergistic integration of Conditional Recurrent Variational Autoencoder (C-RVAE) for learning “typical strategic progressions” and constrained counterfactual inference for alignment.

The introduction of the dimensionless deviation metric, |*Δ*|, proved to be a pivotal innovation. It enabled a dual-view analysis that not only identifies which gameplay elements are most critical but also pinpoints when decisive divergences occur. Our quantitative evaluation on professional tournament datasets validated the framework’s robustness, revealing that strategic deviations in the tested corpus are predominantly rooted in the early game (averaging 8.4% of match duration) and are frequently driven by technology timing gaps. The counterfactual module demonstrated high effectiveness, achieving a similarity improvement of over 90% by correcting these specific divergences. Furthermore, the expert human evaluation confirmed the practical value of the system, awarding high scores for Factual Fidelity (4.6/5.0) and Causal Coherence (4.3/5.0) to the automatically generated narratives, forging a verifiable bridge between abstract vector data and actionable coaching advice.

Despite these contributions, several limitations open avenues for future research. First, while the learned archetypes are interpretable, they remain data-driven summaries; incorporating an expert-in-the-loop mechanism could further ground their strategic semantics. Second, our “counterfactual” analysis provides plausible “what-if” scenarios under feasibility constraints (via projection operators) but does not strictly prove causality in the counterfactual sense used in causal inference literature. Lastly, our narrative generation currently relies on a structured template system to ensure fidelity; while effective, it lacks the stylistic flexibility of modern Large Language Models (LLMs).

Future work will proceed along three main directions. First, we aim to evolve the representation learning stage from the current C-RVAE to hierarchical or goal-conditioned architectures to better capture long-term strategic planning beyond immediate feature correlations. Second, we plan to integrate LLMs conditioned on the structured evidence produced by TRACE, combining the rigorous factual accuracy of our metric-based templates with the nuanced reasoning capabilities of generative AI. Finally, we will focus on developing a fully interactive coaching system where users can pose specific queries (e.g., “What if I had prioritized air upgrades?”), transforming TRACE from a static analytical pipeline into a dynamic strategic dialogue tool.

## Data Availability

The raw data supporting the conclusion of this article is available in: 10.57760/sciencedb.32642.

## Appendix A

### Implementation details

To ensure the reproducibility of the TRACE framework, this appendix specifies the exact data preprocessing steps, model architectures, and algorithmic parameters used in the experimental evaluation. The raw gameplay data was first down sampled to a fixed time step of Δt=1 second (approximately 22.4 game loops). Regarding the “robust scaling” mentioned in the methodology, we implemented a global Z-score standardization (StandardScaler) rather than a min-max approach. The mean μ and standard deviation σ were computed across the entire training corpus to preserve the relative magnitude of features between early and late game phases, ensuring that outliers in individual matches do not skew the global feature space. To address the “light smoothing” requirement for noise suppression, we applied a uniform 1D convolution filter (moving average) with a window size of w=5 steps to the calculated distance metrics. This specific smoothing parameter was chosen to filter out high-frequency jitter caused by micro-management inputs while preserving the sharp gradients characteristic of strategic shifts.

The archetypal path learning module utilizes a Conditional Recurrent Variational Autoencoder (C-RVAE). The encoder consists of a bidirectional LSTM with a hidden dimension of 256, while the decoder employs a unidirectional LSTM of the same size. The latent space dimension was set to 64, balancing representation capacity with bottleneck regularization. The model was trained using the Evidence Lower Bound (ELBO) objective, with the condition vector c encoding the specific matchup type. Optimization was performed using the Adam optimizer with a learning rate of $10^{-3}$ and a batch size of 32 over 100 epochs, utilizing early stopping based on validation reconstruction error.

For the counterfactual interface, the optimization objective relies on a weighting matrix W, which is defined as a diagonal matrix where each diagonal element $W_{\mathrm{ii}}=1/\sigma_{i}^{2}$ corresponds to the inverse variance of feature i. This weighting scheme ensures that features with naturally larger ranges (e.g., mineral income) do not dominate those with smaller ranges (e.g., base count). The domain-specific feasibility constraints (Equation 4) are enforced through a convex combination strategy during trajectory generation. Since both the actual trajectory x and the learned archetypal path A represent valid game states within the physics engine’s constraints, their interpolated counterfactual ${\hat{x}}_{t}=(1-\alpha_{t})x_{t}+\alpha_{t}A_{t}$ inherently satisfies basic feasibility bounds, such as non-negativity and resource conservation, without requiring computationally expensive external solvers. The divergence detection threshold was dynamically set to the maximum of a base value (0.6 standard deviations) and the local signal statistics (median plus half a standard deviation) within the smoothed window.
